# Complex Formation of Phytic Acid With Selected Monovalent and Divalent Metals

**DOI:** 10.3389/fchem.2020.582746

**Published:** 2020-09-23

**Authors:** Gregor Marolt, Ema Gričar, Boris Pihlar, Mitja Kolar

**Affiliations:** Department of Analytical Chemistry, Faculty of Chemistry and Chemical Technology, Universitly of Ljubljana, Ljubljana, Slovenia

**Keywords:** phytic acid, inositol hexaphosphate, metal complexes, magnesium, zinc, iron, copper, phytate

## Abstract

The formation of metal complexes with phytic acid is a complex process that depends strongly on the metal-to-ligand molar ratio, pH value and consequent protonation level of the phytate ligand as well as accompanying side reactions, in particular metal hydrolysis and precipitation of the formed coordination compounds. In the present work, the potentiometric titration technique was used in combination with a detailed analysis of the equivalent point dependencies for selected biologically relevant monovalent and divalent cations from the groups of alkaline earths and transition metals, namely: Mg(II), Zn(II), Fe(II), Cu(I), and Cu(II) ions. The investigation of complex formation mechanism, the evaluation of the species formed, and the identification of other side reactions was based on the examination of three distinct equivalent points, which were detectable by alkalimetric titrations of phytic acid in the presence of selected metal ions. It has been demonstrated that alkaline earth metals interact with different binding site(s) than the transition metals, and experiments with both oxidation states of copper revealed similar complexing characteristics, which depend mainly on the ionic radius (and not on the ionic charge as initially expected). Quantitative data on phytate complexation, hydroxide formation and complex precipitation are presented herein for all metals studied, including Cu(I), which was investigated for the first time by means of alkalimetric titration.

## Introduction

**Figure 1:**
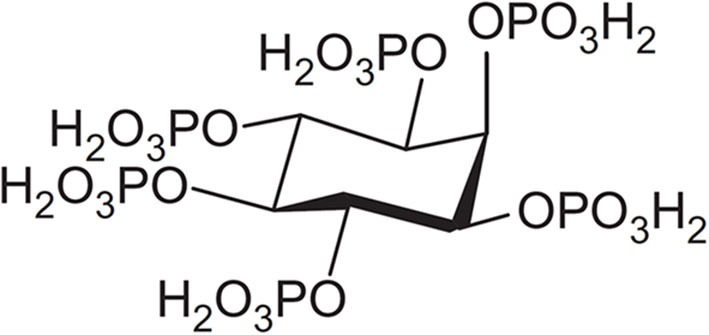
Structure of phytic acid.

Phytic acid (H_12_Phy), a naturally occurring compound found in many biological systems (Sasakawa et al., 1995; Zi et al., 2000), is also known as *myo*-inositol 1,2,3,4,5,6-heksakis(dihydrogen phosphate) and consists of six phosphate esters with two protons per group (see structure in Figure 1), making it a unique molecule with 12 reversibly exchangeable protons that can be released depending on the experimental conditions, i.e., the pH and the presence of metal ions (De Stefano et al., 2004; Crea et al., 2007, 2008; Bretti et al., 2013). Because of its ability to form strong complexes with many metal ions (Evans and Pierce, 1982; Crea et al., 2008), phytic acid, as phytate in its various deprotonated states, is generally considered as an anti-nutrient when present in food (Oatway et al., 2001; Kumar et al., 2010), as it can interfere and thus reduce the bioavailability and consequent absorption of nutrients, including proteins (Cheryan and Rackis, 1980), carbohydrates (Yoon et al., 1984), lipids (Kumar et al., 2010), and minerals (Lopez et al., 2002), such as zinc(II), iron(II/III), calcium(II), magnesium(II), manganese(II), and copper(II) (Nissar et al., 2017). Many papers have also been dealing with antioxidant (Graf and Eaton, 1990) and anti-corrosive (Gao et al., 2014) properties of phytic acid, as well as its environmental (Oatway et al., 2001) and biological (Sasakawa et al., 1995; Vucenik and Shamsuddin, 2003) roles.

These important (biological) properties and effects arise mostly from electrostatic interactions between negatively charged phytate species and metal cations and ability for their chelation (Crea et al., 2008). They have been studied by many researchers using various analytical techniques, including potentiometry (Torres et al., 2005; Šala et al., 2011; Marolt and Pihlar, 2015), spectrophotometry (Heighton et al., 2008), and NMR (Bebot-Brigaud et al., 1999) in combination with computational data analysis (Torres et al., 2008), which has led to numerous series of papers reporting the thermodynamic protonation and formation stability constants of phytic acid and its corresponding metal complexes (De Stefano et al., 2006; Crea et al., 2008). However, many useful information can be derived from these data, but it has been recently shown that a particular attention should be given to the precise standardization of phytic acid prior to measurements, since even a relatively small uncertainty in determination of concentration can cause significant deviations of the derived stability constants using computational methods (Marolt and Pihlar, 2015). As a supplement to the thermodynamic data, it has been shown that the detailed analysis of the titration curves provides additional information on the formation of metal complexes, particularly when other processes, such as precipitation and metal hydroxide formation, accompany the investigated complexation reactions (Šala et al., 2011; Marolt and Pihlar, 2015).

In the case of multiple oxidation states of metal ions, voltammetric methods have proven to be useful as they can provide complementary data on chelation mechanism, molar ratios, complex stability, and other accompanying processes (Torres et al., 2005). In this regard, cyclic voltammetry with the use of Au and HMDE as working electrodes has recently been applied for the detailed characterization of Fe(II) and Fe(III) phytate complexes (Marolt et al., 2015). The investigation of their reaction mechanism at different pH conditions showed a significantly higher stability as well as the predominance of phytate species with a lower protonation level for coordination compounds with trivalent iron ions compared to the divalent ions.

In this context, the aim of this study was to investigate the interactions between phytic acid and selected monovalent and divalent metal ions from the groups of alkaline earth and transition metals by alkalimetric potentiometric titrations. With the analysis of equivalent point(s) (EPs) dependence on the metal-to-ligand molar ratio, new complex formation data regarding complex stability, binding sites, and precipitation reactions are presented herein for selected biologically relevant metals, namely Mg(II), Zn(II), Fe(II), Cu(I), and Cu(II), in the form of coordination compounds with the phytate ligand. Fundamental titrimetric data represent an important contribution to the complete understanding of phytic acid complex formation mechanism, as well as complementary information to the existing literature, including for Cu(I) phytate coordination compounds, which to our knowledge, have not been investigated before.

## Materials and Methods

Aqueous solution of phytic acid was prepared by weighing the dipotassium salt (K_2_H_10_Phy, Sigma-Aldrich, min. 95%) and dissolving in ultrapure water with resistivity of > 18.2 MΩ/cm (Millipore/MilliQ system). The protonated form of phytate (i.e., H_12_Phy) was obtained by passing the solution over a strong cation exchange resin (Dowex 50WX8) using a 100 mL preparative glass column and ~40 g of resin which was wetted in ultrapure water overnight prior to the experiment. The amount of phytate added for each procedure was calculated considering its initial protonation level as well as the volume (~75 mL) and capacity (1.7 meq/mL) of the wetted cation exchange resin. During the ion exchange procedure the column flow was set to 0.25 mL/min and the total contact time between phytate and resin was 5 h. An additional extension of the contact time (using a reduced flow) did not increase the protonation level of phytate, which was typically 11.5 ± 0.2. A differential alkalimetric standardization approach introduced by Marolt and Pihlar (2015) was used to accurately determine the phytic acid concentration (amount). The potassium, sodium, calcium and magnesium concentrations in the eluate were analyzed with an atomic absorption spectrometer (Varian AA240), and were all below 2.0 × 10^−6^ M after single ion exchange procedure. MgCl_2_ · 6H_2_O (Carlo Erba, p. a. grade), ZnCl_2_ (Merck, p. a. grade), FeSO_4_ (Sigma, p. a. grade), CuCl, and CuCl_2_ (both Merck, p. a. grade) salts were used without further purification. Cu(I) and Fe(II) solutions were prepared in 0.20 M HCl which was previously deaerated with argon (purity 5.0) in order to prevent the formation of metal hydroxides and the oxidation of the metal ions with oxygen. Both solutions were kept in a dark room with continuous argon flow and used max. 24 h after the preparation. All titration curves were corrected for the contribution of the added HCl amount, subsequently.

The potentiometric titrations were carried out at 25 ± 1°C by automatic titrator Metrohm 799 GPT Titrino, equipped with a 20 mL burette (accuracy of the increment ± 0.5 μL), a carbon dioxide trap, a magnetic stirrer (Metrohm) and a combined glass electrode (Metrohm 6.0234.100, pH 0–14), calibrated with five buffer solutions (pH 2.00, 4.00, 7.00, 10.00, and 12.00, Merck). Titrations and calculations of titration curve derivatives were performed by TiNet 2.4 software (Metrohm). The titrant was carbonate-free 0.10 M NaOH, which was prepared from concentrated NaOH (Carlo Erba, p. a. grade), dissolved in ultrapure water, deaerated with argon, and standardized weekly with potassium hydrogen phthalate primary standard (Merck). The initial volume of the solution in titration cell was set to 50.0 mL for all experiments.

In the case of Cu(I) and Fe(II), all solutions used, including phytic acid and titrant, were deaerated hitherto and the experiments were carried out in a tightly sealed titration (originally electrochemical) cell (Metrohm) with a constant argon flow. After performing the titration, a sample (1.0 mL) of the final solution was taken using 1 mL micropipette and transferred to a separate electrochemical cell with previously deaerated electrolyte (0.1 M KNO_3_). Metrohm Autolab PGSTAT302N potentiostat was used in combination with 663 VA Stand, equipped with hanging mercury drop electrode (HMDE) as working, Ag/AgCl as reference, and Pt as counter electrode. Cyclic voltammogram (CV) was recorded starting from the open current potential (OCP) toward more negative potential in order to check for the absence/presence of oxidized metals, i.e., Cu(II) and Fe(III) ions, which could be possibly formed during the titration in case oxygen would have entered the cell. However, in all the studied cases, no such oxidized products were detected, confirming the stability of the reduced forms of selected metals, i.e., Cu(I) and Fe(II), which were thus kept in the cell throughout the titration procedure.

## Results and Discussion

### Phytic Acid Titration and General Acid-Base Properties

As stated above, phytic acid in its fully protonated form (H_12_Phy) consists of 12 exchangeable protons, and the protonation equilibrium of phytate (Phy^12−^) is usually considered in the literature (De Stefano et al., 2004; Torres et al., 2005; Crea et al., 2008) by the following reaction:

(1) $$\begin{array}{l} {\text{H}}^{+}+{\text{H}}_{i-1}{\text{Phy}}^{\left(12-i+1\right)-}\overset{K_{i}^{\text{H}}}{↔}{\text{H}}_{i}{\text{Phy}}^{\left(12-i\right)-}, \end{array}$$

where Phy^12−^represents the completely deprotonated form of phytic acid and the index *i* is the number of protonation step, 0 ≤ *i* ≤ 12. At constant ionic strength and temperature one can define the apparent protonation constant *K*_*i*_^H^, which is given according to the equilibrium (1):

(2) $$\begin{array}{l} {\text{K}}_{i}^{\text{H}}=\frac{\left[{\text{H}}_{i}{\text{Phy}}^{\left(12-i\right)-}\right]}{\left[{\text{H}}^{+}\right]\left[{\text{H}}_{i-1}{\text{Phy}}^{\left(12-i+1\right)-}\right]} \end{array}$$

**Figure 2:**
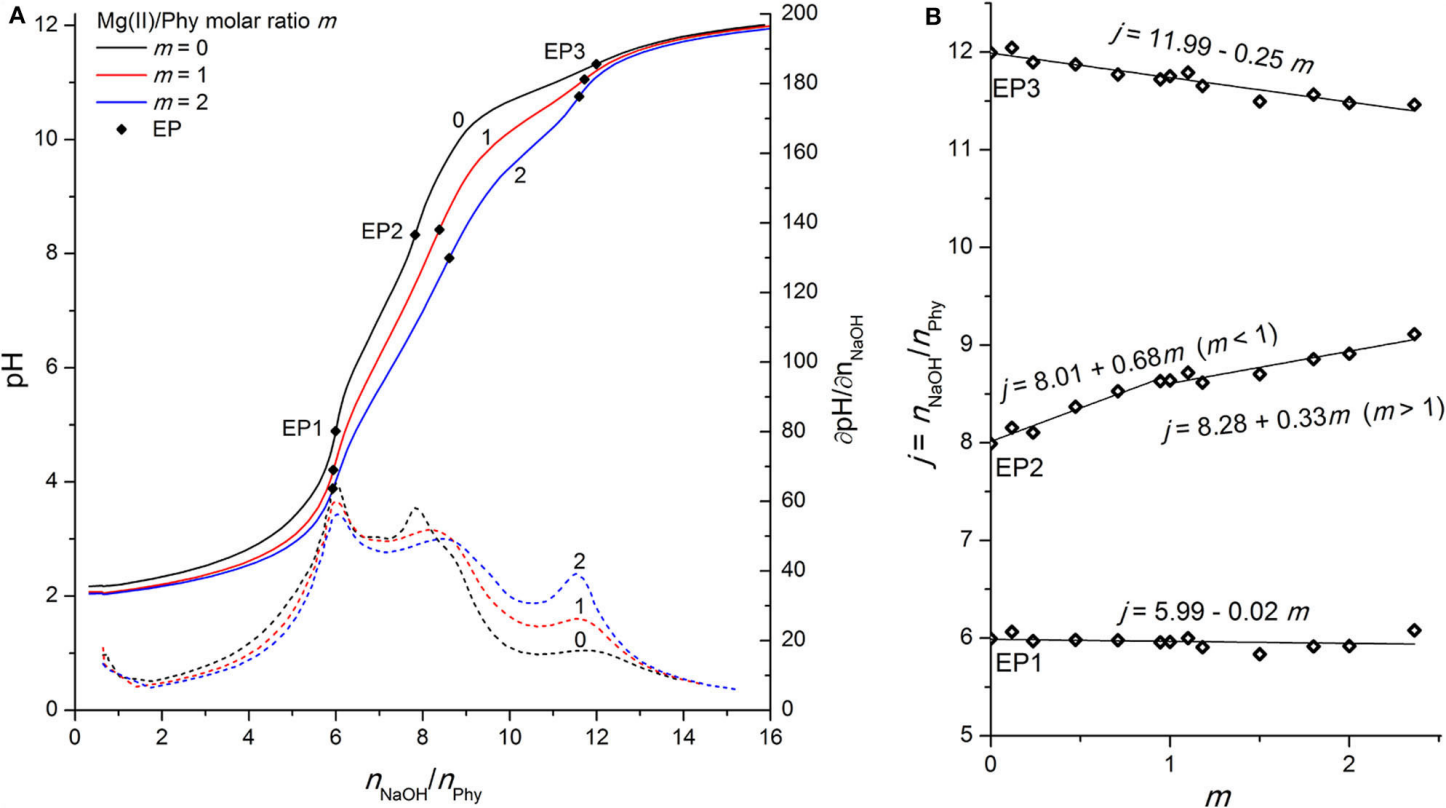
**(A)** Titration of 0.1421 mmol H_12_Phy with 0.1038 M NaOH at various molar ratios *m:* Mg(II)/Phy = 0:1, 1:1, and 2:1, accompanied by corresponding derivatives ∂pH/∂*n*(NaOH); **(B)** Equivalents of NaOH per amount of phytic acid *j* consumed for individual equivalent point at different molar ratios *m* from part **(A)**.

Large sets of thermodynamic speciation data, given as protonation constants of phytate at different experimental conditions, namely ionic strengths and temperatures, can be found in numerous publications (De Stefano et al., 2003a; Bretti et al., 2013) and collected in the review (Crea et al., 2008). However, in order to demonstrate the actual process of alkalimetric titration it is also suitable to write the general form of deprotonation of phytic acid:

(3) $$\begin{array}{l} {\text{H}}_{12}\text{Phy}+j{\text{OH}}^{-}↔{\text{H}}_{12-j}{\text{Phy}}^{j-}+j{\text{H}}_{2}\text{O}, \end{array}$$

where *j* represents the molar ratio between the added titrant and the phytate: *j* = *n*(NaOH)/*n*(Phy), and can also be expressed as deprotonation step number. As reported before (Marini et al., 1981; Evans et al., 1982; Veiga et al., 2014; Marolt and Pihlar, 2015), the titration curve of phytic acid exhibits relatively complicated characteristics due to the high number of (de)protonation steps, and usually only some of them (2–4 deprotonation steps, depending on experimental conditions) are expressed as distinct EPs because of small differences between their corresponding *K*_*i*_^H^ values and consequently smaller pH steps for given EPs. Therefore, the use of titration curve derivatives ∂pH/∂*n*(NaOH) is necessary for the determination of EPs. A similar behavior can be observed in Figure 2 (at molar ratio *m* = 0), where three EPs have been distinguished for the titration of phytic acid in the absence of multivalent metal ions. EP1, EP2, and EP3 appear around pH 4, pH 8, and pH 10.5 and correspond to the 6th, 8th, and 12th deprotonation step (i.e., *j* = 6, 8, and 12), respectively (Marolt and Pihlar, 2015):

(4) $$\begin{array}{l} {\text{H}}_{12}\text{Phy}+6{\text{OH}}^{-}↔{\text{H}}_{6}{\text{Phy}}^{6-}+6{\text{H}}_{2}\text{O} \end{array}$$

(5) $$\begin{array}{l} {\text{H}}_{6}{\text{Phy}}^{6-}+2{\text{OH}}^{-}↔{\text{H}}_{4}{\text{Phy}}^{8-}+2{\text{H}}_{2}\text{O} \end{array}$$

(6) $$\begin{array}{l} {\text{H}}_{4}{\text{Phy}}^{8-}+4{\text{OH}}^{-}↔{\text{Phy}}^{12-}+4{\text{H}}_{2}\text{O} \end{array}$$

On this basis, the exchangeable phytic acid protons can be divided into three groups according to their acidity (Marolt and Pihlar, 2015) and used for further analysis of the titration curves in the presence of metal ions:

- First group of the 6 most acidic protons with ${log\;K}_{7-12}^{\text{H}}$ < 2.7 ± 0.1, released before EP1
- Second group of 2 intermediate protons with an average ${log\;K}_{5-6}^{\text{H}}$ of 5.6 ± 0.6, released between EP1 and EP2
- Third group of the 4 most strongly bound protons with ${log\;K}_{1-4}^{\text{H}}$ > 8.0 ± 0.1, released between EP2 and EP3.

Another process, that can also accompany deprotonation and make the titration curve of the phytic acid even more complicated, is the pH-dependent inversion of the molecule from the equatorial (1a5e) to the axial (5a1e) orientation of 6 phosphate groups of phytate (Torres et al., 2008). This molecular switch is reported to appear between pH 9.0 and pH 9.5 and has been well-studied by Brigando et al. (1995) and by Veiga et al. (2014) using ^31^P NMR titrations and molecular modeling in combination with NMR spectroscopy, respectively. When dealing with the analysis of phytic acid titration curves and EPs dependencies in combination with the addition of metal ions, all this knowledge is a prerequisite for the correct investigation of the formation of corresponding complexes.

### Phytic Acid Interactions With Mg(II) Ions

Due to the high affinity of negatively charged phytate to various cations, the presence of mono- and multivalent metal ions affects the shape of the phytic acid titration curves and can therefore be used to identify the undergoing processes and the complex formation. As shown in Figure 2A, the addition of Mg(II) affects the shape of the whole titration curve of phytic acid, particularly in the range between EP1 and EP3, similar to the observations for Ca(II) interactions described in our previous work (Marolt and Pihlar, 2015). The decrease of pH before EP1 in the presence of Mg(II) compared to the pH values recorded for phytic acid alone is the result of interactions between added metal ions and phytic acid already before EP1 is reached and consequently an increased acidity of the first (i.e., most acidic) group of protons of phytic acid. A similar behavior can also be observed for the recorded pH values at EP1, while the consumption of NaOH at EP1 remains unchanged for all investigated molar ratios, showing that the amount of released protons before EP1 does not change with the addition of Mg(II) ions. In the following region, between equivalent points EP1 and EP3, one can observe more pronounced effect, since the EP2 is moved toward greater titrant consumption upon gradual addition of the Mg(II) concentration and consequently increased metal-to-ligand molar ratio: *m* = *n*(M^z+^)/*n(*Phy). This means that Mg(II) ions compete with protons for binding sites of the phytate ligand between pH 4 and pH 10 and therefore increase the acidity of the second group of protons (*vide infra*), which causes the pH drop for a given amount of added NaOH. Another consequence is also the gradual shift of EP2 and EP3 while the position of EP1 remains constant, as stated before. This can be clearly seen on the Figure 2B which shows the dependence of titrant equivalent consumption *j* on the molar ratio *m* for each EP observed. A linear slope value of +0.68 is observed at EP2 for *m* ≤ 1, and the slope is halved to +0.33 for *m* > 1. This shows that when phytate is in excess, around 2 mol of protons are released per 3 mol of added Mg(II), and “only” 1 mol of protons per 3 mol of Mg(II) when the amount of Mg(II) in the solution is higher than the amount of phytate, indicating a change in the reaction mechanism after the first equivalent of Mg(II) is bound to the ligand. The position of EP1 remains constant while EP3 is shifted toward a slightly lower consumption of NaOH, which means that a complete deprotonation of phytic acid at EP3 is reached earlier, presumably due to the precipitation of phytate at pH > 10 in the form of the Mg(II) complex, according to the literature data (Evans and Pierce, 1982; Veiga et al., 2006). The EP3 derivative values increase as the final deprotonation step becomes more pronounced at higher metal-to-ligand molar ratios. This is due to the well-known decrease in *K*_*i*_^H^ values and the increased acidity of the last (third) proton group with the highest affinity for phytate, which has also been reported for phytic acid titrations in the presence of other alkaline earths (Martin and Evans, 1986; Marolt and Pihlar, 2015) and alkali metals (Li et al., 1989a,b; De Stefano et al., 2003b).

### Phytic Acid Interactions With Zn(II) Ions

**Figure 3:**
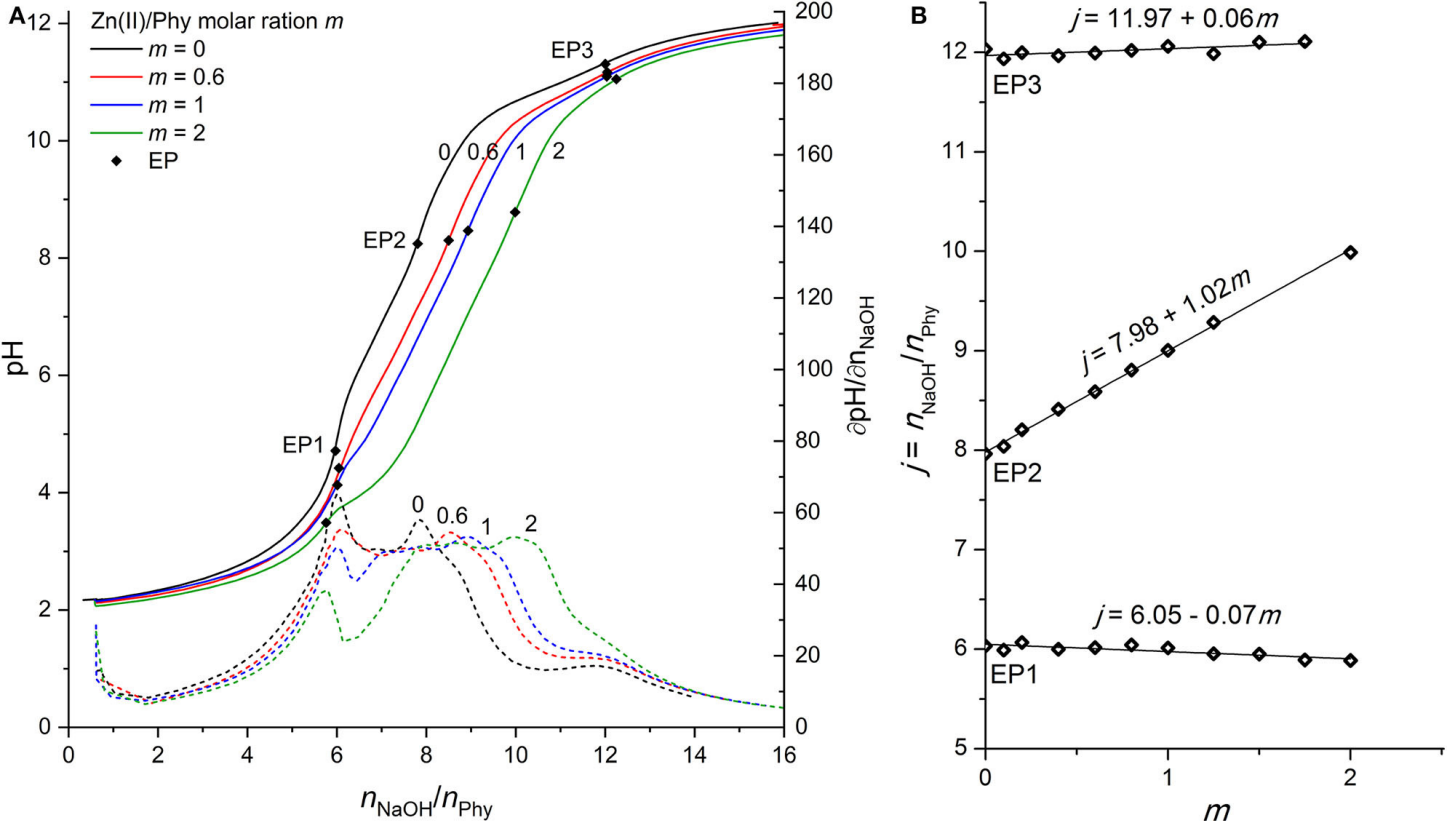
**(A)** Titration of 0.1389 mmol H_12_Phy with 0.1032 M NaOH at various molar ratios *m:* Zn(II)/Phy = 0:1, 0.6:1, 1:1, and 2:1, accompanied by corresponding derivatives ∂pH/∂*n*(NaOH); **(B)** Equivalents of NaOH per amount of phytic acid *j* consumed for individual equivalent point at different molar ratios *m* from part **(A)**.

As shown in Figure 3A, the complexation of Zn(II) ions with phytic acid exhibits similar behavior to that of Mg(II), as the pH of the titration curve in the region before EP1 as well as between EP1 and EP3 is shifted toward lower values and the EP2 is shifted toward a higher NaOH consumption with increasing molar ratio *m*. This can again be explained by the complexation of metal ions competing for binding sites of the phytate ligand and consequently increasing the acidity of the first, second, and third group of protons which are therefore released earlier. However, compared to Mg(II) the slope of the titrant equivalent consumption *j* at EP2 to the molar ratio *m* is higher (+1.02) and constant for all molar ratios studied (see Figure 3B), which means that 1 mol of protons is released per 1 mol of added Zn(II). This is an indication that Zn(II) ions are attached to a different binding site than Mg(II) and other alkaline earth metals, since a similarly lower slope (+0.59) of EP2 has also been reported for Ca(II) (Marolt and Pihlar, 2015). While EP2 is shifted, EP3 remains constant for all molar ratios studied, which means that the final deprotonation step appears at the same position, indicating the absence of precipitation of Zn(II) phytate complexes in this pH range, in contrast to the case of Mg(II). Although the NaOH consumption remained constant at EP1, a detailed analysis of the titration curves in Figure 3A shows that the pH of titration curve before and at EP1 decreases with increasing *m*, which is an evidence of metal complex formation already at low pH where phytate species with a higher level of protonation are present in solution and in accordance with reports of complex formation below pH 6 for Zn(II) ions (Torres et al., 2005). Furthermore, at higher excess of Zn(II) ions, e.g., at *m* = 2, a significantly lower pH slope in the region closely after the EP1 can be observed, as well as the decreased values of corresponding derivatives, shown in Figure 3A. This could again be explained by the occurrence of metal complexes with a higher level of protonation, i.e., [ZnH_6_Phy]^4−^ (Torres et al., 2005), and by higher stability constants of Zn(II) complexes with phytic acid (Crea et al., 2008). Hereby, it is worth mentioning that due to the relatively low values of the derivative curves around EP2 and consequently less distinct maxima, 3 to 5 replicates were derived for each molar ratio studied in order to provide an accurate determination of EP2, which was placed at the same position within the experimental error <2% using the software equipment.

### Phytic Acid Interactions With Fe(II) Ions

Phytic acid complexes with iron are known for their high stability and important biological role, including antioxidant properties, as reported in many publications (Lee et al., 1998; Vucenik and Shamsuddin, 2003; Oomah et al., 2008; Quirrenbach et al., 2009). The complexes are strongly dependent on the pH, the metal-to-ligand molar ratio, and the corresponding stability constants of trivalent iron complexes are significantly higher than those of divalent iron (Torres et al., 2005). Coordination compounds of iron phytates and their antioxidant role in preservation chemistry (Kolar et al., 1998, 2005; Strlič et al., 2001; Wagner and Bulska, 2003) have also been extensively studied by our research group, especially Fe(III) ions using various analytical techniques, including potentiometric (Šala et al., 2011; Marolt and Pihlar, 2015) and NMR titrations (Bebot-Brigaud et al., 1999; Mali et al., 2006), while “only” voltammetric methods have recently been used (Marolt et al., 2015) to study the Fe(II)/Fe(III) redox couple in the presence of phytic acid, providing important information about the electrochemical mechanism that is strongly dependent on the pH and the consequent protonation level of the ligand. Therefore, the aim of this work was to evaluate the Fe(II) phytate acid-base properties using alkalimetric titration and to obtain a deeper understanding of the iron phytate complex formation mechanism.

**Figure 4:**
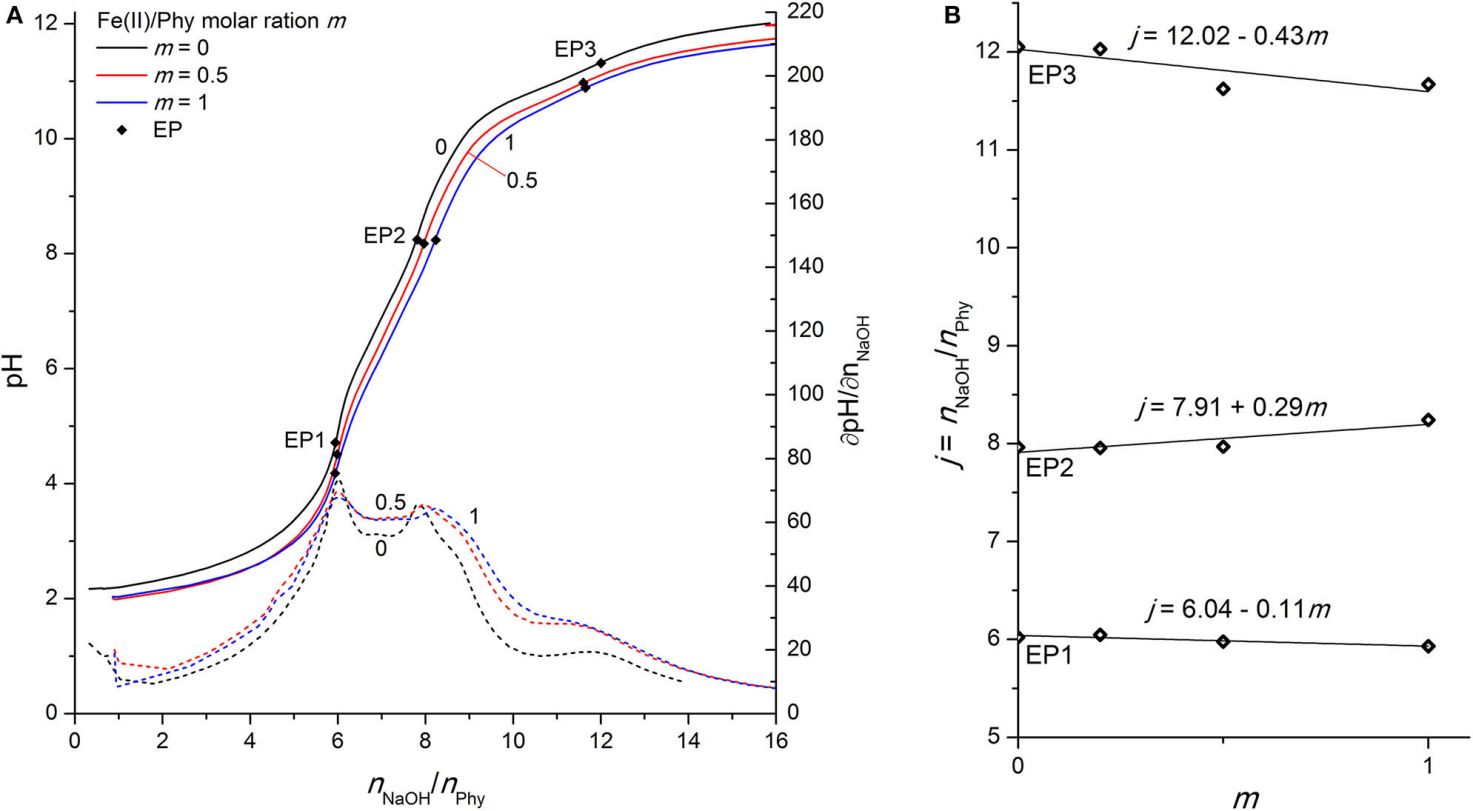
**(A)** Titration of 0.1528 mmol H_12_Phy with 0.1002 M NaOH at various molar ratios *m:* Fe(II)/Phy = 0:1, 0.5:1, and 1:1, accompanied by corresponding derivatives ∂pH/∂*n*(NaOH); **(B)** Equivalents of NaOH per amount of phytic acid *j* consumed for individual equivalent point at different molar ratios *m* from part **(A)**.

As stated above, the interactions of phytic acid with trivalent iron are several orders of magnitude greater (Torres et al., 2005) than those with divalent iron, which can also be confirmed in Figure 4A, where only a minor differentiation of the phytic acid titration curve is observed upon the addition of Fe(II) ions. While the position of EP1 remains almost constant (the corresponding slope is −0.11), EP2 and EP3 shift at an increased molar ratio *m* with an average slope of +0.29 mol and −0.43 mol of titrant per 1 mol of added Fe(II), respectively (see Figure 4B). These values differ significantly from the values reported for the Fe(III) ions with slopes of +1.67, +2.26, and +1.94 for EP1, EP2, and EP3, respectively (Marolt and Pihlar, 2015). This is a clear identification of different phytic acid complex formation mechanisms between divalent and trivalent iron and is in good agreement with the reports of voltammetric investigation of Fe(II)/Fe(III) phytates (Marolt et al., 2015). Fe(II) ions do not form coordination compounds with highly protonated phytate species before EP1 (below pH 5), and also have much less influence on the acidity of the second and third group of phytic acid protons, based on the observed EP2 dependence. Due to the smaller positive charge and the larger ionic radius of Fe(II) compared to Fe(III), divalent ions sustain significantly weaker binding interactions with phytate and therefore have less effect on the acidity of phytic acid protons. From the analysis and comparison with the titration curves of other divalent ions discussed above, it can be deduced that Fe(II) ions form less stable complexes under given conditions than Mg(II) and Zn(II), as also reported by Torres et al. (2005). A negative slope of EP3 implies that Fe(II) phytate complexes precipitate above pH 9, which was also observed during the titrations already at low metal-to-ligand molar ratios. Hence, only experiments up to *m* = 1 were conducted for Fe(II) due to the large amounts of white-colored precipitate. However, contrary to the titrations with trivalent iron (Marolt and Pihlar, 2015), divalent did not undergo the metal hydrolysis under the experimental conditions applied in this work.

### Phytic Acid Interactions With Cu(II) Ions

**Figure 5:**
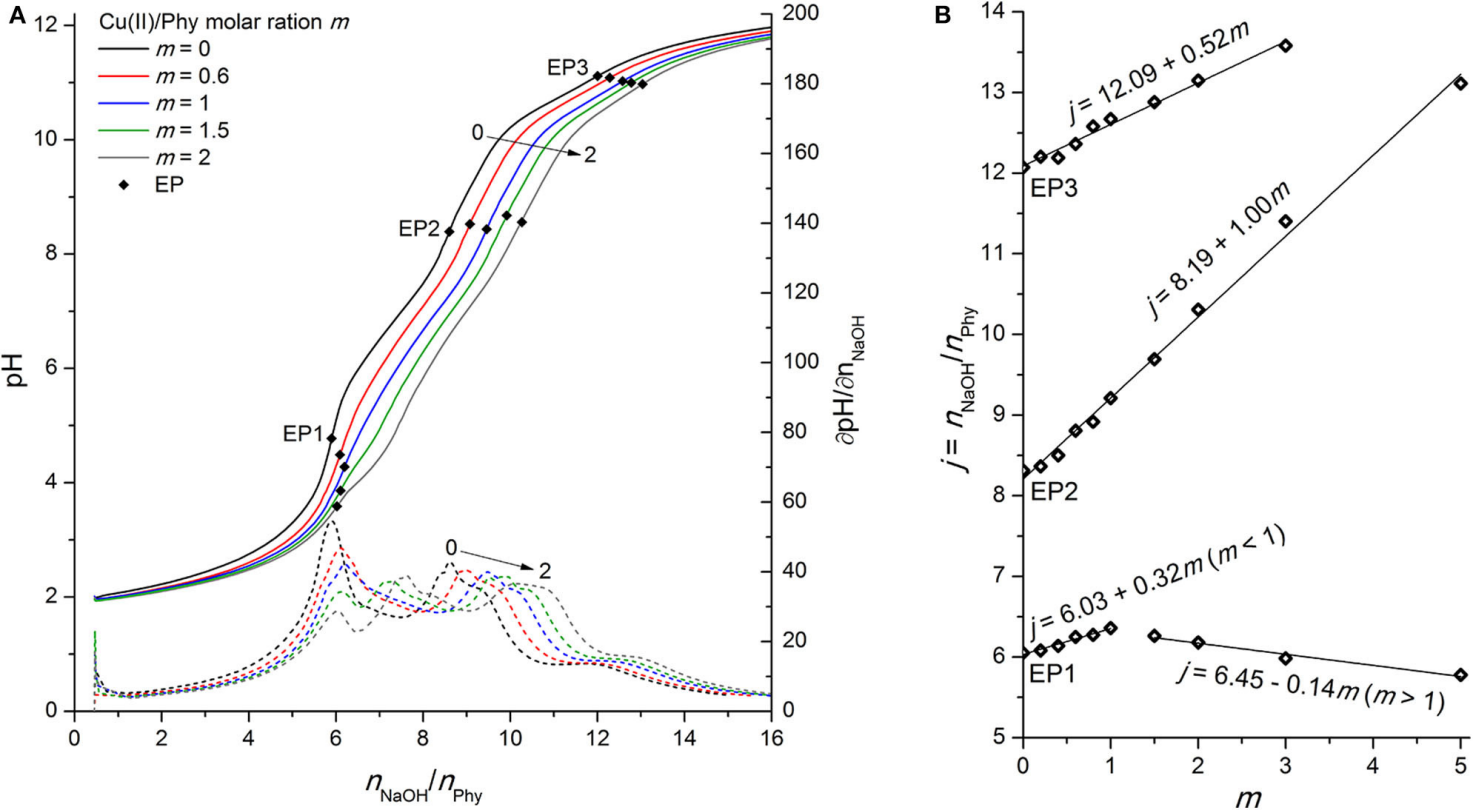
**(A)** Titration of 0.0951 mmol H_12_Phy with 0.1178 M NaOH at various molar ratios *m:* Cu(II)/Phy = 0:1, 0.6:1, 1:1, 1.5:1, and 2:1, accompanied by corresponding derivatives ∂pH/∂*n*(NaOH); **(B)** Equivalents of NaOH per amount of phytic acid *j* consumed for individual equivalent point at different molar ratios *m* from part **(A)**.

In contrast to the Fe(II) experiments, the titration curve of phytic acid in the presence of Cu(II) ions exhibits more pronounced alternations over the entire titration range, as shown in Figure 5A. As in the case of Zn(II), the pH value before and at the EP1 decreases with increasing metal-to-ligand molar ratio, which can be explained by the onset of complex formation already before EP1. Cu(II) ions therefore compete with the first group of protons for binding sites of phosphate groups and increase their acidic properties, which is also confirmed by the increased titrant consumption at EP1 for *m* ≤ 1 with the slope value of +0.32, meaning around 1 mol of protons is released per 3 mol of added Cu(II) in the region before EP1 (Figure 5B). Similarly, the positive shift of EP2 with an average slope of +1.00 is due to the complexation of Cu(II) with the phytate ligand, which indicates that 1 mol of protons is exchanged by 1 mol of added Cu(II) in the pH range of the second proton group with intermediate acidity. On the other hand, the increased consumption of NaOH with the slope of + 0.51 at the final equivalent point (EP3), corresponding to the neutralization of the last (12th) phytate proton, is an identification of the formation of stable Cu(II) hydroxide species, which are also observed as a light blue precipitate that was formed in the titration cell at pH 9 and above, according to the reactions from the literature (Martell and Smith, 1977):

(7) $$\begin{array}{l} {\text{Cu}}^{2+}+2{\text{OH}}^{-}\overset{K_{2,1}}{↔}{\left[\text{Cu}{\left(\text{OH}\right)}_{2}\right]}_{\left(s\right)} \end{array}$$

(8) $$\begin{array}{l} {\text{Cu}}^{2+}+3{\text{OH}}^{-}\overset{{\;}^{\text{​​​​}K_{3,1}}}{↔}{\left[\text{Cu}{\left(\text{OH}\right)}_{3}\right]}^{-} \end{array}$$

(9) $$\begin{array}{l} 2{\text{Cu}}^{2+}+2{\text{OH}}^{-}\overset{K_{2,2}}{↔}{\left[{\text{Cu}}_{2}{\left(\text{OH}\right)}_{2}\right]}^{2+} \end{array}$$

with considerably high values of the corresponding stability constants: *log K*_2,1_ = 12.8, *log K*_3,1_ = 14.5, and *log K*_2,2_ = 17.8 (Martell and Smith, 1977), confirming that the soluble Cu(II)-hydroxo species and Cu(OH)_2_ precipitate are predominant forms of copper(II) under alkaline conditions, as discussed by Cuppett et al. (2006). The titration of Cu(II) with NaOH in the absence of phytic acid (not shown), revealed the non-stoichiometric molar ratio between Cu(II) and OH^−^ ions, indicating the formation of mixed Cu(II) hydroxide species. The equivalent point for this reaction was found at pH 8.4, which is close to the range of the second EP of phytic acid titration curve, indicating that the shift of EP2 could also be partly due to the occurrence of Cu(II) hydroxo species.

A detailed analysis of the dependence of NaOH consumption on the metal-to-molar ratio on Figure 5B shows a positive shift of EP1 with a slope of +0.32 until the equimolar ratio between Cu(II) and phytate is reached (*m* ≤ 1) and a negative slope of −0.14 hereinafter (*m* > 1). This is most likely due to the altered reaction mechanism for *m* > 1, e.g., the formation of polynuclear Cu(II) phytate species, such as [Cu_2_H_5_Phy]^3−^, which were also observed by Crea et al. (2007) and Vasca et al. (2002). However, another reason could be the precipitation of the complex before reaching EP1 (and the resulting dissolution due to the positive slope of EP2 and EP3 in the whole range of molar ratios studied), but is very unlikely at such a low pH, and there was also no visible evidence of any precipitate formation during titration under acidic pH conditions, which supports the first explanation.

### Phytic Acid Interactions With Cu(I) Ions

**Figure 6:**
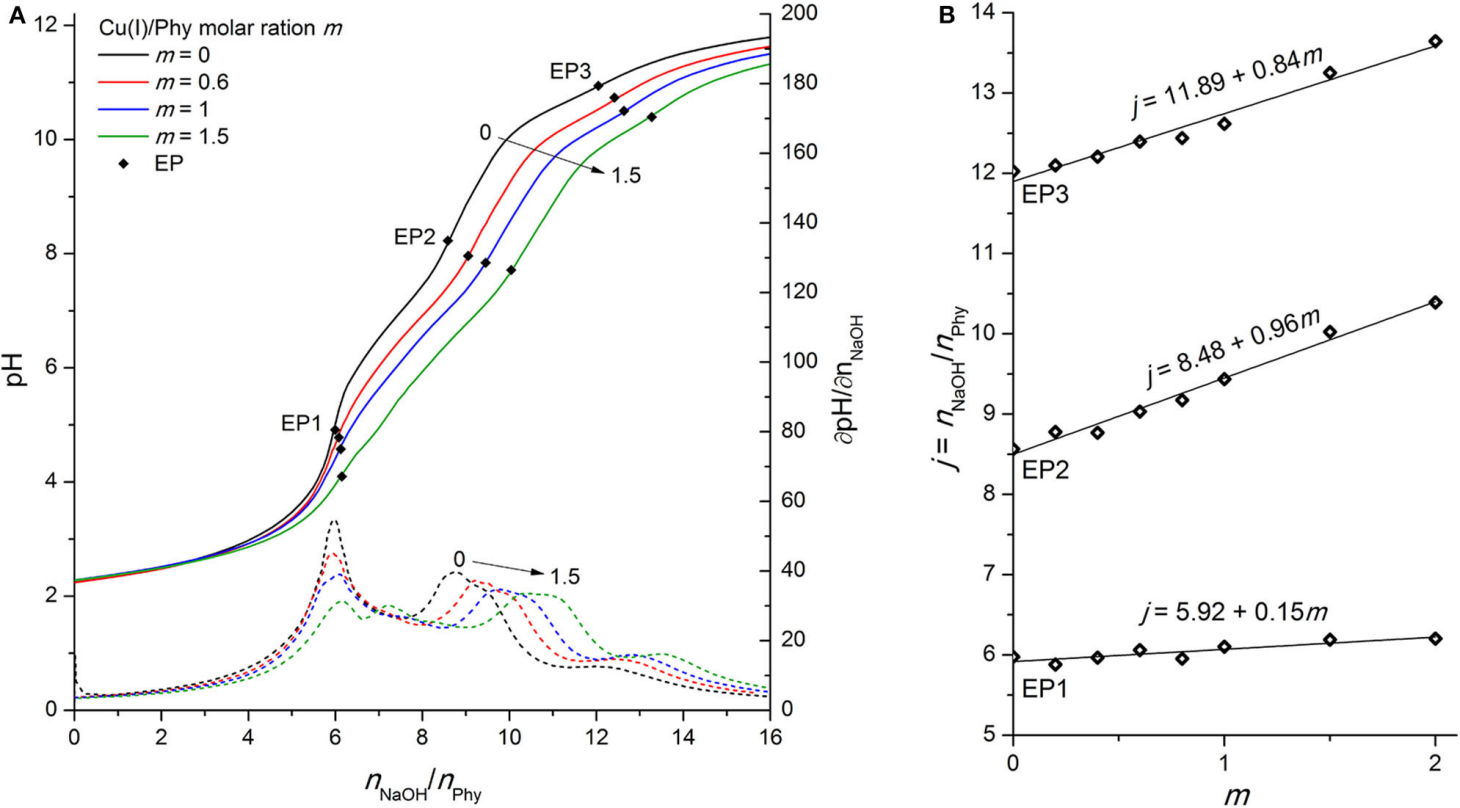
**(A)** Titration of 0.1096 mmol H_12_Phy with 0.1105 M NaOH at various molar ratios *m:* Cu(I)/Phy = 0:1, 0.6:1, 1:1, and 1.5:1, accompanied by corresponding derivatives ∂pH/∂*n*(NaOH); **(B)** Equivalents of NaOH per amount of phytic acid *j* consumed for individual equivalent point at different molar ratios *m* from part **(A)**.

In the literature there are not many studies that address the Cu(I) complexes with phytic acid (Li et al., 2013), probably also due to the unstable nature of monovalent copper, which can be rapidly oxidized to divalent state in the presence of dissolved oxygen. Therefore, likewise in the case of Fe(II), all titrations were carried out in a tightly-sealed cell with an inert atmosphere under constant Ar flow and special care was taken to prevent the entry of oxygen at any stage of the experiment. Figure 6A shows the behavior of phytic acid titration curve upon addition of Cu(I) ions, which is similar to that of Cu(II) (see Figure 5A), since the curve and consequent EP shifts can be observed practically throughout the whole titration range.

However, some differences between the two oxidation states can be observed, starting with the constant slope of the consumed NaOH dependence on the added Cu(I) for all studied molar ratios (*m* ≤ 2). This is an indication of “only” one complex formation mechanism in the pH range around EP1, in contrast to the Cu(II) complexes where two types of behavior depending on *m* were found. The lower value of the slope of EP1 [+0.15 vs. +0.32 for Cu(I) vs. Cu(II)] is presumably due to the weaker interactions of the less-positively charged monovalent ion with the most acidic group of phytic acid protons. However, perhaps contrary to one's initial expectations, the slope of titrant consumption at EP2 (+0.96) is very close to that of divalent copper (+1.00), which shows that a similar complex formation mechanism is taking place in the region of the second proton group with intermediate acidity for both oxidation states, regardless the difference of their charge. Considering the similarity of the ionic radius of Cu(I) and Cu(II) with values of 0.77 and 0.73 Å, respectively, it can be assumed that, rather than the total ion charge (or charge density), the ionic radius of transition metal ion is of greater importance for phytic acid complex formation and resulting deprotonation of the ligand, particularly in the intermediate pH range between EP1 and EP3. In support of this, a similar EP2 dependence (with a slope of +1.02) was also observed for Zn(II) ions (see Figure 3B) with similar ionic radius as copper (0.74 Å), while significantly less pronounced shift of EP2 (with slope of +0.29, Figure 4B) was found for much smaller Fe(II) ions (0.61 Å). Furthermore, if we take into consideration the reports of Fe(III) ions (0.55 Å) (Šala et al., 2011; Marolt and Pihlar, 2015), the distinction between titration curves of divalent and trivalent iron can be explained by the difference of ionic radii, which is in contrast to the previously mentioned case of the two oxidation states of copper. Fe(III) revealed a remarkably greater EP2 dependence on *m* with a slope of +2.26 (Marolt and Pihlar, 2015), which is much higher not only in comparison to that of Fe(II) but also to other metals investigated and thus shows the strongest binding interactions with phytate. This is in good agreement with stability constants with several orders of magnitude higher values, which were reported by Torres et al. (2005), as well as with recent findings of the electrochemical study of the Fe(II)/Fe(III) phytate complex mechanism by Marolt et al. (2015). However, in the case of Fe(III), strong binding affinity to the phytate ligand is also due to the electrostatic effects, caused by the higher cation charge of trivalent iron (Crea et al., 2008), which is also the reason for the interaction with the most acidic proton group and the resulting shift of EP1 with a slope of +1.67.

On the other hand, the results of Mg(II) and Ca(II), the latter of which was published previously (Marolt and Pihlar, 2015), do not follow the same trend, since an evidently lower slope of the EP2 position was found in the case of both alkaline earth metal ions. This could be explained by differences in the binding sites of phytate, as an individual group of metals (i.e., alkaline earth metals vs. transition metals) interact with their preferred phosphate position(s) of the ligand (Marolt and Pihlar, 2015) and cause deprotonation of proton(s) with different acidic properties (i.e., *K*_*i*_^H^ values), resulting as a shift of different EPs on the titration curve of phytic acid. For clarification, all discussed EP slopes and ionic radii of the studied metal complexes with phytic acid from previous and this work are summarized in Table 1.

**Table 1:** Ionic radii and slope values of the dependence of the molar ratio *j*^a^ between titrant (NaOH) and phytic acid on the metal-to-ligand molar ratio *m*^b^ at individual equivalent point (EP).

**Metal ion**	**Ionic radius [Å]**	**EP1**	**EP2**	**EP3**	**References**
Mg(II)	0.72	−0.02	+0.68; +0.33^c^	−0.25^d^	This work
Ca(II)	1.00	+0.01	+0.59	−0.03	Marolt and Pihlar, 2015
Cu(I)	0.77	+0.15	+0.96	+0.84	This work
Cu(II)	0.73	+0.32; −0.14^c^	+1.00	+0.52	This work
Zn(II)	0.74	−0.07	+1.02	+0.06	This work
Fe(II)	0.61	−0.11	+0.29	−0.43^d^	This work
Fe(III)	0.55	+1.67	+2.26	+1.94	Marolt and Pihlar, 2015

a j = n_OH_/n_Phy_;

b m = n_M_/n_Phy_;

c Values given for m ≤ 1 and m > 1, respectively;

d *Observed precipitation*.

As clarly seen from Figure 6A, the final deprotonation step is also shifted to a higher consumption of the titrant at increased concentration of Cu(I) ions with an average slope of +0.84 mol per 1 mol of added copper (see Figure 6B). The higher slope of monovalent compared to divalent copper (+0.52) demonstrates that the Cu(I) undergoes a metal hydrolysis reaction to a greater extent, which was also confirmed by the visual detection of yellow-colored precipitate that was formed in the titration cell during the titration. As a result, a lower concentration of Cu(I) ions is present in chelated form of the phytate complex around EP3, which is detected at pH > 10.5. Titration of Cu(I) ions in the absence of phytic acid (and with the addition of HCl prior to titration to increase the solubility of CuCl salt) revealed the molar ratio of the reaction between hydroxide and monovalent copper; n(OH^−^) : n(Cu(I)) = 3 : 2 (not shown). This indicates the presence of two Cu(I) hydroxo species; [Cu(OH)_2_]^−^ and [Cu(OH)] in the molar ratio 1:1 (Illas et al., 1984). The formation of the latter can also be better described by the following reaction (Martell and Smith, 1977):

(10) $$\begin{array}{l} 2{\text{Cu}}^{+}+3{\text{H}}_{2}\text{O}↔{\text{Cu}}_{2}{\text{O}}_{\left(s\right)}+2{\text{H}}_{3}{\text{O}}^{+}, \end{array}$$

but the logarithmic value of the corresponding reaction constant is considerably low (−14.7) (Martell and Smith, 1977), which means that the Cu_2_O precipitate is stable only under alkaline conditions with sufficiently high concentration of hydroxide ions (and consequently low concentration of H_3_O^+^).

## Conclusions

In summary, the formation of metal complexes with phytic acid is a complex process that depends strongly on the molar ratios, the pH conditions, and the associated protonation level of phytate ligand as well as on the accompanying side reactions, in particular metal ion hydrolysis and precipitation of the formed coordination compounds. In this work, the potentiometric titration technique was applied in combination with a detailed analysis of the detected equivalent point dependencies on the metal-to-ligand molar ratios for the selected divalent and monovalent cations. The differences in the slope values of NaOH consumption observed at individual EPs suggest that the alkaline earth metals, i.e., Mg(II), discussed here, and previously studied Ca(II) (Marolt and Pihlar, 2015), presumably bind to different phosphate groups than the transition metals, i.e., Zn(II), Cu(I), Cu(II), and Fe(II). It is important to note that this could be also due to the formation of metal-phytate species with different stoichiometry. Although several NMR titration studies have been conducted on (metal-)phytate systems (Brigando et al., 1995; Bebot-Brigaud et al., 1999; Mali et al., 2006; Šala et al., 2011), to the best of our knowledge, only Champagne et al. (1990) reports the same preferential phytate binding site for Ca(II) and Cu(II) ions, which appears to be the P5 phosphate group or position between P5 and P4 or P6 phosphate groups. However, no specifications about the proton release has been provided. In general, the metal-phytate interactions are of high complexity, as the multivalent metals typically interact with two or three phosphate groups simultaneously and can therefore cause the release of proton(s) and/or favorize the inversion of phytate from equatorial to axial conformation (Torres et al., 2008; Veiga et al., 2014).

In the present work, it was shown that Fe(II) has a significantly distinct complex formation mechanism than the rest of the transition metal group, which is due to the well-known specific binding interactions between phytic acid and iron ions in general, as well as the accompanying precipitation process which is more pronounced compared to other metal complexes investigated in this work. Experiments with different oxidation states of copper revealed similar complexation characteristics for both monovalent and divalent metal ions, which supports the explanation that the reaction mechanism depends primarily on the ionic radius and is independent of the total charge and/or charge density of the complexed metal ions. A similar reaction mechanism has been also found for Zn(II) with an ionic radius comparable to that of the two copper oxidation states, while a considerably smaller Fe(II) and Fe(III) ions exhibit contrasting behavior (Marolt and Pihlar, 2015). The latter one exhibits significantly higher EP2 dependence and thus the strongest binding interaction with phytate among the metals investigated, which could be confirmed by the reported stability constants with several orders of magnitude higher values (Torres et al., 2005) as well as by the findings of our previous electrochemical study of the Fe(II)/Fe(III) phytate complex mechanism (Marolt et al., 2015).

Based on the present work, other phytic acid coordination compounds with alkaline earth metals, e.g., Sr(II) and Ba(II) ions, and/or transition metals of different oxidation states and ionic radii are to be investigated in the future, possibly by potentiometric and/or NMR titrations in combination with voltammetric techniques in order to confirm and/or obtain a deeper insight into the phytate complex formation mechanism(s) and understanding of the interactions between phytic acid and metal ions.

## Data Availability Statement

The raw data supporting the conclusions of this article will be made available by the authors, without undue reservation.

## Author Contributions

GM and MK: conceptualization. GM, EG, BP, and MK: methodology. GM and EG: validation, formal analysis, and investigation. GM: writing—original draft preparation. GM, BP, and MK: writing—review and editing. BP and MK: supervision. All authors contributed to the article and approved the submitted version.

## Conflict of Interest

The authors declare that the research was conducted in the absence of any commercial or financial relationships that could be construed as a potential conflict of interest.
